# Factors associated with blood pressure control in Swedish primary care patients with hypertension: a cross-sectional study

**DOI:** 10.1080/02813432.2025.2524366

**Published:** 2025-06-30

**Authors:** Niklas Brodin, Moa Wolff, Beata Borgström Bolmsjö, Veronica Milos Nymberg, Peter Nymberg, Susanna Calling

**Affiliations:** ^a^ Office for Primary Care, Skåne University Hospital, Malmö, Sweden; ^b^ Department of Clinical Sciences, Center for Primary Health Care Research, Lund University, Malmö, Sweden

**Keywords:** Hypertension, blood pressure, primary health care, antihypertensive agents, combination drug therapy

## Abstract

**Purpose:**

This study aimed to investigate the factors associated with blood pressure control in a primary healthcare population with hypertension.

**Materials and methods:**

We used baseline data from a recent Swedish randomized controlled trial where 400 patients diagnosed with hypertension from 10 primary health care centers were included. The participants underwent blood pressure measurements, blood sampling and completed questionnaires on quality of life, physical activities, tobacco- and alcohol use, medication, and comorbidities. Logistic regression analyses were used to estimate odds ratios (OR) and 95% confidence intervals (CI) for factors associated with blood pressure control (<140/90 mmHg).

**Results:**

The mean age of the participants was 69 years. The results showed blood pressure control in 41% of the cases. The factors that had the highest ORs for achieving blood pressure control were previous myocardial infarction (OR 2.44; CI 1.08–5.53), diabetes diagnosis (OR 2.26; CI 1.31–3.88), and use of ≥2 blood pressure medications (OR 1.62; CI 1.07–2.46). Family history of hypertension was negatively associated with blood pressure control (OR 0.29; CI 0.38–0.88) (univariate analyses).

**Conclusions:**

Our study found an association between the use of ≥2 antihypertensive medications and blood pressure control. Despite current treatment guidelines for hypertension, the use of single-drug therapy remains common. By shifting from single drug to combination therapy, focusing on patients with a family history of hypertension and those without comorbidities, the proportion achieving blood pressure control could increase significantly.

**Trial registration:**

ClinicalTrials.gov (NCT04407962).

## Introduction

Hypertension is the strongest modifiable risk factor for ischemic heart disease, stroke, and kidney failure [1], and is defined as repeated clinic blood pressure (BP) measurements ≥140/90 mmHg. With a prevalence of around 30–45% in the general population in Europe, hypertension is the leading risk factor for mortality [2]. Recent studies have suggested that ∼45% of patients with diagnosed hypertension in Western high-income countries do not achieve BP control and thus have a significantly increased risk of future cardiovascular disease [3]. The situation is similar in Sweden [4].

Hypertension is typically detected and treated in primary care. About 12% of consultations in primary care concern high BP, which makes it the number one diagnosis for clinical visits in primary care worldwide [5].

In Sweden, primary care is provided by tax-funded private and public primary health care centers (PHCCs), which manage the majority of patients with hypertension. Typically, patients with hypertension have an annual check-up and prescription renewal with their general practitioner (GP). However, due to a shortage of GPs, some parts of Sweden have nurse-led teams at the PHCCs that treat patients with hypertension with support from the GP and from local delegation guidelines [6].

International guidelines for hypertension management have faced criticism for burdening primary care services with their high requirements for primary prevention of cardiovascular risk factors. Primary preventive interventions might also divert attention from treating the most clinically relevant risk factor: namely BP itself [7].

Research on factors associated with BP control (<140/90 mmHg) has shown that ethnic origin, having health insurance, and the number of BP check-ups per year significantly influence BP management outcomes [8,9]. Previous studies investigating factors associated with successful and unsuccessful BP control in primary care have been limited and produced conflicting outcomes. For example, in one Malaysian study, comorbidity and ≥3 BP medications were associated with poor BP control, whereas in a larger Canadian study, comorbidity and combination therapy for high BP were associated with favorable BP control [10,11]. Hence, it is still uncertain which factors are associated with BP control, particularly in a primary care setting.

If we can identify success factors for BP control in patients diagnosed with hypertension we can, conversely, pinpoint patient groups at risk of not achieving BP control, allowing for additional efforts to be specifically directed toward them.

The purpose of this study was to examine which factors are associated with BP control in a cohort of Swedish primary care patients diagnosed with hypertension.

## Materials and methods

### Study design

We conducted a cross-sectional study using baseline data derived from a Swedish randomized controlled trial examining the effects of motivational text messages on BP and other cardiovascular risk factors in a primary care population with hypertension (the Primary Care Usage of Health Promoting Messages, PUSHME study, registered 2020-05-28 at ClinicalTrials.gov, NCT04407962).

### Participants and recruitment

Between September 2020 and December 2022, eligible patients were identified *via* electronic charts search at 10 primary health care centers (PHCCs) in southern Sweden (the regions Skåne, Kronoberg, Västra Götaland, and Stockholm). Inclusion criteria were patients aged 40–85 years and with diagnosed hypertension (ICD-10: I10). Patients were identified at each PHCC and exported to an Excel list. The list was then randomized, and patients were invited sequentially from the top until the target number of participants had been reached at each PHCC (aim: 50 per center).

At the PHCCs, the identified patients were invited to participate in the study *via* postal mail. Within two weeks, they were contacted by phone by a study nurse who provided additional information about the study and, if applicable, scheduled a baseline visit at the patient’s PHCC. Written informed consent was collected at the baseline visit.

Patients were excluded if their baseline visit BP was ≥180/110 mmHg (grade 3 hypertension) or if their systolic BP (SBP) was <120 mmHg. The reason for exclusion in these cases was that, in the RCT, interventions involving physical activity were not considered suitable for patients with grade 3 hypertension, and we did not expect any health benefits from lowering BP below 120 mmHg. Other exclusion criteria were serious illnesses associated with a short life expectancy (<1 year) and anticipated difficulties in adhering to the study protocol, such as cognitive impairment.

The baseline visits were conducted by a research nurse at each PHCC and consisted of a physical examination and completion of a self-reported questionnaire to collect information on current medication, medical history, family history of hypertension, years since hypertension diagnosis, previous or current use of cigarettes or snus*, alcohol consumption, physical activity level, self-rated health, health-related quality of life, and educational level. *Snus is an oral nicotine product consisting of moist powder tobacco and is typically used by placing it under the upper lip.

The study design and procedures for the PUSHME study were approved by the Swedish Ethical Review Authority (Dnr 2019-06361). Informed consent was obtained from all participants, and the research was performed in accordance with the Declaration of Helsinki. Monitoring was performed at each study site by a clinical trial nurse to ensure that the study protocol was followed accordingly.

### Study measures

BP was measured using the brachial cuff method according to the guidelines of the European Society of Hypertension, i.e. in a sitting position after 5–10 min of rest [12]. We used Electronic BP monitors that were procured for healthcare centers in southern Sweden (Boso Medicus). Mean BP was calculated from two measurements (mean of three readings if the first and second readings differed by >5 mmHg). An independent dichotomized variable was created as ‘controlled BP’ or ‘high BP’, where controlled BP was defined as SBP 120–<140 mm Hg and DBP <90 mm Hg, and high BP was set to ≥140 mmHg SBP or ≥90 mmHg DBP according to WHO’s definition [13].

Waist circumference was measured between the iliac crest and the lowest rib in a standing-up exhaled phase. BMI results were grouped into normal weight (≤24.99 kg/m^2^), overweight (25.00–29.99 kg/m^2^), and obese (≥30 kg/m^2^) [13].

The assessment of self-rated health was made using the question ‘How would you rate your general health?’ with a 5-point Likert scale response: very good, good, fair, poor, very poor [14]. In the analysis of self-rated health, the response options ‘very good’ and ‘good’ were recoded into ‘good self-rated health’ and the remaining response options into ‘poor self-rated health’. Health-related quality of life was evaluated using the validated EQ5D-5L questionnaire, which measures impairment levels across five dimensions: mobility, self-care, usual activities, pain/discomfort, and anxiety/depression [15]. Family history of hypertension was defined by a ‘yes’ response to the question: ‘Do you have any biological relatives who currently have or have previously had high blood pressure?’. Tobacco and oral nicotine usage was dichotomized as ‘Currently using’ or ‘Not using or previously used’. Alcohol consumption was measured as the number of drinks per week (one drink equals 12 grams of alcohol). For men, 0–4 drinks were categorized as ‘Low consumption’, 5–14 as ‘Moderate consumption’, and ≥15 as ‘High-risk consumption’. For women, 0–4 drinks were categorized as ‘Low consumption’, 5–9 as ‘Moderate consumption’, and ≥10 as ‘High-risk consumption’ [16]. For the logistic regression models, the alcohol variables were dichotomized into ‘Low’ or ‘Moderate/high’.

Physical activity was measured in minutes per week of exercise (e.g. running or fitness class, i.e. physical exercise that makes you breathless) and non-exercise activity (e.g. going for a walk or slow cycling). Validated questions from the National Board of Health and Welfare were used [17]. Exercise and non-exercise activity were converted into ‘activity minutes’ using the formula activity minutes = 2*exercise + 1*non-exercise activity [17] (see Supplementary Figure 1). Patients at or above the recommended activity level were categorized as ‘physically active’, while patients below the recommended levels were categorized as ‘sedentary’ [17].

Chronic ischemic heart disease is comprised of the following categories: current or previous angina pectoris, prior myocardial infarction, and coronary angioplasty or bypass surgery. This composite variable was used in the multiple regression model because these patient groups are considered equivalent in terms of medication and cardiovascular risk.

### Medications

Current medications were self-reported by the study participants and confirmed by the study nurse during the baseline visit. The total number of drugs and the number of BP drugs per patient were registered, as well as any use of lipid-lowering medication. The BP-lowering medication was categorized according to ATC codes (ATC: Anatomical Therapeutic Chemical Classification System) into eight different groups [18].

Blood samples were collected for analyses of HbA1c, total cholesterol, Low-Density Lipoprotein (LDL) cholesterol, and High-Density Lipoprotein (HDL) cholesterol. Non-HDL-cholesterol was calculated as total cholesterol minus HDL-cholesterol.

### Statistics/data analysis

Data were analyzed using IBM SPSS Statistics 27 (IBM Corp., Released 2020, Armonk, NY, USA). Differences between groups were calculated using two-tailed *t*-test for continuous variables, Mann–Whitney *U* test for nonparametric variables, and Chi-Square test for categorical variables. Normality was tested using Kolmogorov-Smirnov and Shapiro-Wilk scores with a cut-off limit of *p* = 0.05, in combination with reviewing histogram plots. An EQ5D-5L Index Value Calculator, developed by the EuroQol Group, was used to cal­culate the EQ5D-5L index value [19]. Univariate logistic regression analyses were conducted for relevant variables that demonstrated significant differences between controlled and uncontrolled BP in Chi-Square tests. Using ≥2 BP drugs was considered the most interesting factor associated with BP control, as it may be modifiable by medical treatment. Therefore, the subsequent multiple logistic regression analyses were performed to study the relationship between ≥2 BP drugs and controlled BP using three models with additional adjustments for potential confounders: (1) age and sex, (2) family history of hypertension, and (3) comorbidities (diabetes and chronic ischemic heart disease).

## Results

**Figure 1.**
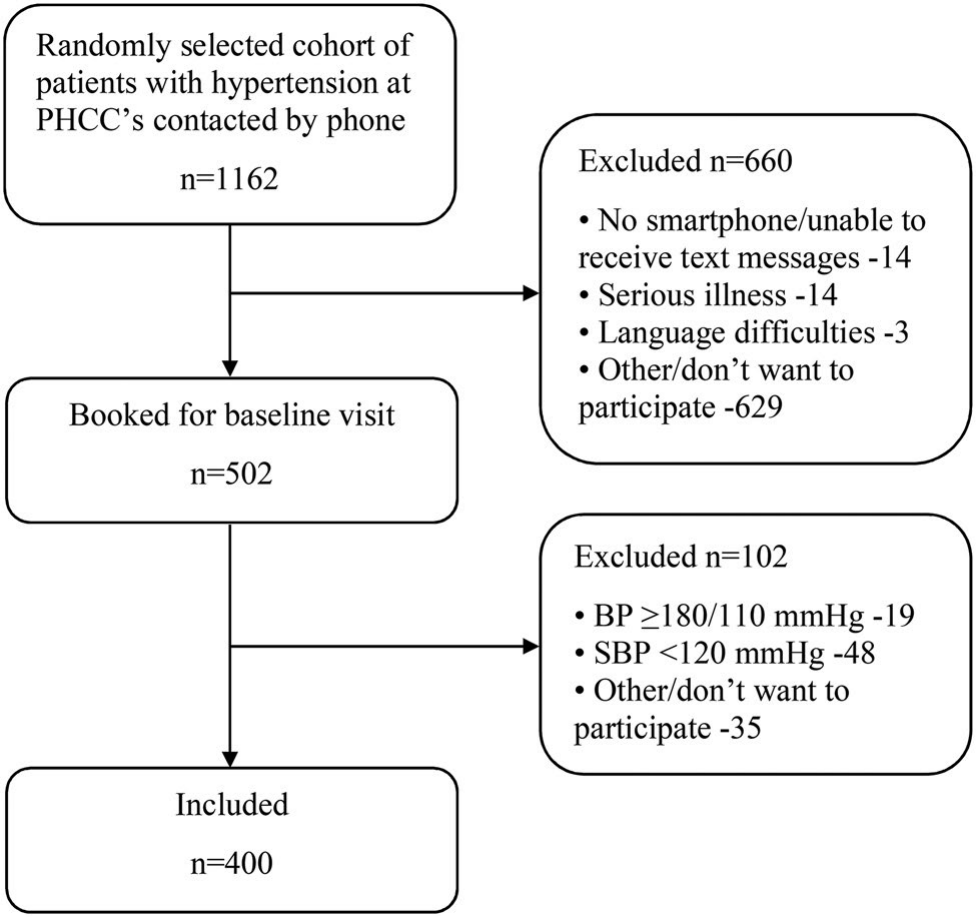
Flowchart of recruitment.

A total of 1162 invited patients were contacted by phone by a study nurse. Out of these, 43% (502 patients) accepted participation and were invited to a baseline visit. At the baseline assessment, 48 participants were excluded due to low SBP (<120 mmHg), 19 due to high BP (SBP ≥180 mmHg and/or DBP ≥110), and an additional 35 participants withdrew for various reasons or other unspecified causes. See Figure 1 for the flowchart of recruitment.

The sample of 400 included participants consisted of 209 men and 191 women aged 41–86 years of age (mean 69 years). Demographics and clinical characteristics of participants are presented in Table 1. Most of the patients were overweight (BMI ≥25 kg/m^2^).

**Table 1.** Demographics and clinical characteristics.

Variable	Total	Controlled BP 120–<140/<90	High BP ≥140/90	*p*
	*N* = 400	*n* = 164	*n* = 236	
Age, median (IQR)	70 (13)	70 (12)	69 (14)	0.366
Women, *n* (%)	191 (47.8)	73 (44.5)	118 (50)	0.280
BMI, kg/m^2^, median (IQR)	27.9^a^ (6.4)	28.4 (7.5)	27.7 (6.1)	0.069
BMI grouped, *n* (%)				0.284
<25	104 (26.0)	41 (25.2)	63 (26.7)	
25–29.9	168 (42.0)	63 (38.7)	105 (44.5)	
≥30	127 (31.8)	59 (36.2)	68 (28.8)	
Waist women, cm, mean ± *SD*	97.2 ± 13.3^a^	97.9 ± 14.3	96.7 ± 12.6	0.569
Waist men, cm, mean ± *SD*	106.4 ± 11.8	107.9 ± 12.2	105.3 ± 11.5	0.108
HbA1c, median (IQR) (mmol/mol)	38 (6)	39 (7)	38 (6)	**0.011**
Non-HDL cholesterol, median (IQR) (mmol/L)	3.3 (1.5)	3.2 (1.6)	3.4 (1.5)	0.151
Drug treatment				
Total drugs, median (IQR)	4 (4)	4 (3)	4 (4)	**0.043**
BP drugs, median (IQR)	2 (1)	2 (2)	2 (1)	**0.017**
Using ≥2 BP drugs, *n* (%)	244 (61.0)	111 (67.7)	133 (56.4)	**0.022**
Using lipid-lowering drugs, *n* (%)	182 (45.5)	81 (49.4)	101 (42.8)	0.193

BMI: body mass index; BP: blood pressure; IQR: interquartile range.

Chi-Square test for categorical variables, Mann–Whitney *U* test for nonparametric variables, two-tailed *t*-test for continuous variables. Level of significance: *p* < 0.05. Significant *p*-values presented in bold.

^a^Missing weight, height and waist circumference of one female patient. Nonparametric values as displayed as median (IQR). Normally distributed variables are displayed as mean ± *SD*.

The BP control criteria (i.e. 120–<140/<90 mmHg for this population) were met by 41% of participants (164/400), indicating that 59% (*n* = 236) had SBP ≥140 mmHg and/or DBP ≥90 mmHg. When accounting for the 67 patients excluded at baseline because of low SBP (<120 mmHg) and grade 3 hypertension (≥180/110 mmHg), the proportion of patients reaching BP-control (<140/90 mmHg) was instead 45% (212/467). In total, 61% of the patients had ≥2 BP medications, i.e. 39% had ≤1 BP medication. Participants with ≥2 BP medications were more likely to have well-controlled BP (*p* < 0.05), and this finding remained significant even when patients with comorbidities (diabetes and ischemic heart disease) were excluded (*p* < 0.05) (Supplementary Table 1). Participants diagnosed with hypertension for more than five years were more likely to use ≥2 BP medications (*p* < 0.05).

The proportion of patients reaching BP control was higher in the group with snus consumption as well as in the group without a family history of high BP (*p* < 0.05) (Table 2). Among the snus users, nearly half (47%) were former smokers, compared to 27% among those who had never used snus.

**Table 2.** Patient characteristics according to questionnaire.

Variable	Total	Controlled BP 120–<140/<90	High BP ≥140/90	*p*
	*N* = 400	*n* = 164	*n* = 236	
	*n* (%)*	*n* (%)*	*n* (%)*	
Family history of hypertension	263 (65.8)	96 (58.5)	167 (70.8)	**0.011**
Smoking				0.609
Current smoker	17 (4.3)	5 (3)	12 (5.1)	
Previous smoker	126 (31.5)	52 (31.7)	74 (31.4)	
Snus				**0.010**
Currently using snus	30 (7.5)	19 (11.6)	11 (4.7)	
Women’s alcohol consumption				0.571
Moderate	23 (12.0)	8 (11.0)	15 (12.7)	
High	5 (2.6)	3 (4.1)	2 (1.7)	
Men’s alcohol consumption				0.540
Moderate	70 (33.5)	34 (37.4)	36 (30.5)	
High	6 (2.9)	2 (2.2)	4 (3.4)	
Sedentary	238 (59.5)	94 (57.3)	144 (61)	0.458
Previous stroke	18 (4.5)	9 (5.5)	9 (3.8)	0.427
Current or previous angina pectoris	18 (4.5)	7 (4.3)	11 (4.7)	0.852
Previous myocardial infarction	26 (6.5)	16 (9.8)	10 (4.2)	**0.028**
Diabetes	64 (16.0)	37 (22.6)	27 (11.4)	**0.003**
Current or previous aortic aneurysm	3 (0.8)	0 (0)	3 (1.3)	0.147
Coronary angioplasty or bypass surgery	35 (8.8)	17 (10.4)	18 (7.6)	0.340
Chronic ischemic heart disease^§^	42 (10.5)	20 (12.2)	22 (9.3)	0.357
Upper secondary or higher education	291 (72.8)	115 (70.1)	176 (74.6)	0.325
>5 years with hypertension diagnosis	271 (67.8)	109 (66.5)	162 (68.6)	0.646
Good SRH (good and very good)	280 (70.0)	119 (72.6)	161 (68.2)	0.351
SRH graded 1–100, median (IQR)	78 (15)	79 (16)	77 (15)	0.289
EQ5D index (0–1), median (IQR)	0.916 (0.162)	0.922 (0.142)	0.916 (0.138)	0.161

SRH: self-rated health; IQR: interquartile range.

*n (%) unless stated otherwise. Nonparametric values as displayed as median (IQR). Chi-Square test for categorical variables, Mann–Whitney *U* test for nonparametric variables, two-tailed *t*-test for continuous variables. Level of significance: *p* < 0.05. Significant *p*-values presented in bold.

^§^Chronic ischemic heart disease is a composite of the following categories: current or previous angina pectoris, previous myocardial infarction, and coronary angioplasty or bypass surgery.

The prevalence of diabetes or a prior history of myocardial infarction was higher in the group that achieved BP control (*p* < 0.05). The type of BP medication used did not have a significant impact on whether BP control was achieved or not (Supplementary Table 2). We found no significant difference in BP control based on level of alcohol consumption, self-assessed health, or quality of life (self-rated health, EQ5D).

Univariate logistic regression models revealed statistically significant associations between BP control and history of myocardial infarction (OR 2.44; CI 1.08–5.53), prevalent diabetes (OR 2.26; CI 1.31–3.88), and the use of ≥2 BP medications (OR 1.62; CI 1.07–2.46). Family history of hypertension was negatively associated with BP control (OR 0.29; CI 0.38–0.88) (Supplementary Table 3). The use of ≥2 BP medications remained statistically significantly associated with BP control after adjusting for age and sex (model 1, OR 1.58; CI 1.04–2.41), and family history of hypertension (model 2, OR 1.55; CI 1.01–2.36). However, the significance disappeared after adding comorbidities (diabetes and chronic ischemic heart disease) to the analysis (model 3, OR 1.39; CI 0.90–2.14) (Table 3).

**Table 3.** Multiple logistic regression for controlled BP.

Variable	Label	Model 1	Model 2	Model 3
		OR (CI)	OR (CI)	OR (CI)
No of BP drugs used	0–1	1 (ref)	1 (ref)	1 (ref)
	≥2	**1.58** (1.04–2.41)	**1.55** (1.01–2.36)	1.45 (0.94–2.22)
Family history of HT	Yes		1 (ref)	1 (ref)
	No		**1.57** (1.02–2.44)	**1.57** (1.01–2.45)
Diabetes	No			1 (ref)
	Yes			**2.03** (1.16–3.53)
Chronic IHD^§^	No			1 (ref)
	Yes			1.14 (0.58–2.25)

HT: hypertension; IHD: ischemic heart disease.

Model 1: Adjusted for age and sex.

Model 2: Adjusted for age, sex, and family history of hypertension.

Model 3: Adjusted for age, sex, family history of hypertension, and comorbidities (diabetes and IHD).

Significant ORs are presented in bold.

^§^Chronic IHD is a composite of the following categories: current or previous angina pectoris, previous myocardial infarction, and coronary angioplasty or bypass surgery.

## Discussion

Our study found that the use of ≥2 BP medications was significantly associated with BP control (120–<140/<90 mmHg) in this population of Swedish primary care patients diagnosed with hypertension. The association remained after adjustments for age, sex, and family history of hypertension, but disappeared after additional adjustments for comorbidity (diabetes and chronic ischemic heart disease), likely due to overadjustment as patients with comorbidities often have ≥2 BP medications. In line with this, the association between ≥2 BP medications and BP control remained when patients with diabetes and chronic ischemic heart disease were excluded from the analysis. Furthermore, several classes of medications for diabetics contribute to lowering BP without being classified as BP medications (e.g. SGLT2 and GLP1A). Additionally, the threshold for BP control is lower for diabetics, which makes adjustments for this group particularly difficult to interpret. The hypertension guidelines in Europe from 2019 advise an initial use of two antihypertensive drugs in most patients with hypertension [12]. This recommendation is supported by studies that highlight the effectiveness of starting with dual combinations in overcoming two common barriers in clinical practice that hinder BP reduction: therapeutic inertia (the failure to intensify treatment when hypertension is not adequately controlled) and low adherence to the prescribed treatment regimen [20–22]. Clinicians may refrain from initiating or intensifying antihypertensive medication due to various reasons, such as time constraints, concerns about potential side effects, and uncertainty about a patient’s out-of-clinic BP [23].

Patients with reported family history of hypertension were less likely to have controlled BP. This was a robust result that persisted even after adjustment for potential confounders. A family history of high BP has long been established as a risk factor for hypertension [24]. Hence, physicians should prioritize treatment with a minimum of two antihypertensive medications and closely monitor patients with a family history of hypertension.

Our findings, which indicate an association between the presence of diabetes or previous myocardial infarction and BP control, respectively, are in line with a previous Canadian cohort study [11]. The association of comorbidities and BP control could potentially be explained by a ‘health scare effect’, in which a heightened perception of the treatment’s significance and implicitly an improved adherence (by both patients and physicians) often follows a significant event, like a myocardial infarction. Yet, contradicting this assumption is the fact that chronic ischemic heart disease, which might also affect adherence, did not show a significant association with BP control. While this appears contradictory at first glance, it may reflect differences in clinical attention and treatment routines following acute *versus* chronic events.

In our study, 41% of primary care patients with hypertension achieved BP control (120–<140/<90 mmHg). This is a somewhat larger proportion compared to data from a recent Swedish study where only 35% of the primary care population with hypertension achieved controlled BP [25]. However, according to a review from 2021, the proportion of patients achieving controlled BP in high-income Western countries is better than in our study—54% for men and 59% for women [3]. One reason for this discrepancy may be the prolonged strained situation in Swedish primary care with a shortage of GPs and low doctor-patient continuity, which has been described in a Swedish government report. In a society with an aging population and increasing demands on primary care, patients with ‘only’ hypertension and few medications may be perceived as lower priority and, as a result, receive less monitoring and treatment. Our findings suggest that this approach may lead to poorly controlled blood pressure and consequently missed opportunities for early prevention. Antihypertensive combination therapy was mainly observed among those with longstanding hypertension. Considering this, alongside the fact that 39% of our study participants were receiving ≤1 BP medication, it raises the question of whether an overly cautious treatment strategy is being applied in newly diagnosed patients. Also, it highlights the importance of further exploring the reasons behind limited adherence to treatment guidelines.

In contrast to previous research findings, we did not observe any association between BP control and lifestyle factors, such as physical activity, waist circumference, and alcohol consumption in our study [26,27]. Snus was the only lifestyle factor which showed a significant association with BP control. This contrasts with previous larger studies indicating that high BP is more common in snus users [28,29]. It is therefore difficult to draw reliable conclusions from our findings, as more research would be needed to explain such an association. The relationship between lifestyle factors and BP levels is easier to study in larger epidemiological studies or randomized studies with lifestyle interventions.

Our study has several strengths. It stands out compared to most comparable studies by having up-to-date primary care data on many factors that influence cardiovascular risk, including blood tests, heritability, self-rated health, levels of alcohol consumption, and physical activity. Thanks to the broad inclusion criteria and recruitment of patients from diverse regions, the study provides a fair representation of individuals with hypertension in Swedish primary care. The reliability of measurements was very good, as each PHCC was provided with the same BP measurement devices, and the measurements were conducted according to standard procedures by study nurses specially trained for this specific study.

One of this study’s limitations involves its cross-sectional design, which does not allow for establishing a causal relationship between BP control and its associated factors. The exclusion of patients with SBP <120 mmHg from the study is a limitation and means that we lack information about this specific group, and it likely generates an overestimation of the proportion of uncontrolled BP. On the other hand, we know that the lowering of SBP to <120 mmHg does not result in significant advantages regarding cardiovascular risk and should be avoided in most patients [30]. Therefore, the BP treatment of patients with SBP <120 mmHg is less relevant to our aim of identifying factors for successful BP control. Since the study population was recruited from an RCT investigating the effects of motivational text messages on BP and other cardiovascular risk factors, we cannot rule out the possibility of selection bias, with more motivated individuals potentially being overrepresented. Smoking also raises the question of selection bias. The prevalence of current smokers in our study population was only 4.3%, which contrasts with previous research indicating a smoking prevalence of 12.3% among Swedish individuals with hypertension [31]. Non-smokers might be more prone to participate in the study, hence resulting in a healthier cohort. Moreover, we only measured BP on one occasion. Given that BP varies considerably within individuals over time, repeated home BP measurements or 24-h ambulatory BP would have been more accurate methods to measure the patient’s actual BP and to avoid the impact of white coat hypertension on the results. This is, however, time-consuming and requires a much larger effort from the participants, which could possibly cause more dropouts. Another factor that may have affected the level of BP control in our study was that it took place during the COVID-19 pandemic when a significant proportion of medical visits were replaced by digital appointments or canceled entirely [32]. The study lacks data on medication adherence, which would have been valuable to explore, parti­cularly when comparing patients with and without comorbidities. Self-reported answers *via* questionnaire might affect the validity of this study. Two variables that would have been valuable to include, although challenging to accurately capture through self-reporting in a questionnaire, are dietary patterns and compliance with BP treatment. One limitation of this study is the small sample sizes in the subgroup analyses, which reduces the likelihood of detecting significant differences between groups. Additionally, we used a survey questionnaire to assess factors, such as physical activity and alcohol consumption, which have the usual limitations associated with self-reported data.

## Conclusion

Our study showed that the use of ≥2 BP medications was associated with BP control. On the contrary, patients with a family history of hypertension and patients without diabetes or previous myocardial infarction were less likely to reach BP control. Despite current guidelines emphasizing the importance of combination therapy for BP management, 39% of the patients in our study were receiving ≤1 BP medication. To achieve BP goals and consequently reduce the risk of cardiovascular complications, primary care physicians need to recognize the significance of transitioning away from monotherapy in hypertension, especially among patients with a family history of hypertension and those without comorbidities. Further research is required to investigate the factors influencing adherence and non-adherence to BP treatment and BP treatment guidelines.

## Supplementary Material

Supplementary material 250320.docx

## Data Availability

The data that support the findings of this study are available from the corresponding author, upon reasonable request.
