# Supplementary material for: Factors associated with blood pressure control in Swedish primary care patients with hypertension: a cross-sectional study
Source: Scand J Prim Health Care. 2025 Jun 30;44(1):1–9. doi: 10.1080/02813432.2025.2524366 (PMC12918332; doi:10.1080/02813432.2025.2524366)
Supplement: Supplementary material 250320.docx [file IPRI_A_2524366_SM2301.docx]

| **Supplementary table 1.** Number of BP drugs in patients with controlled vs. high BP. Patients with chronic IHD* and diabetes excluded | | | | | | | | | | |
| --- | --- | --- | --- | --- | --- | --- | --- | --- | --- | --- |
|  | | Total | | | Controlled BP  120-140/<90 | | High BP | |  |  |
| **Variable** | | N=306 | | | n=114 | | n=192 | |  | p |
|  |  |  |  |  |  |  |  |  |  |  |
| Using ≥2 BP drugs, n (%) | | | 175 | (57.2) | 74 | (64.9) | 101 | (52.6) |  | **0.035** |
|  |  |  |  |  |  |  |  |  |  |  |
| BP: blood pressure; chronic IHD*: chronic ischemic heart disease is a composite of the following categories: current or previous angina pectoris, previous myocardial infarction, and coronary angioplasty or bypass surgery. Chi-Square test for categorical variables. Level of significance: p <0.05. Significant p-values presented in bold. | | | | | | | | | | |

| **Supplementary table 2** Factors associated with BP control | | |
| --- | --- | --- |
| **Variable** | **Label** | **OR (CI)** |
| **Diabetes** | No diabetes | 1 (ref) |
|  | Diabetes | **2.26** (1.31-3.88) |
| **MI** | No MI | 1 (ref) |
|  | Previous MI | **2.44** (1.08-5.53) |
| **Family history of hypertension** | No | 1 (ref) |
|  | Yes | **0.29** (0.38-0.88) |
| **No of BP drugs used** | 0-1 | 1 (ref) |
|  | ≥2 | **1.62** (1.07-2.46) |
| Separate univariate logistic regression of variables showing significant difference between controlled and uncontrolled BP (table 1-2). Odds ratio (OR) with 95% confidence intervals for controlled BP. Significant ORs presented in bold.  BP: Blood pressure; MI: myocardial infarction | | |

| **Supplementary table 3.** BP medication for groups with controlled vs. high BP | | | | | | | |
| --- | --- | --- | --- | --- | --- | --- | --- |
|  | Controlled BP  120-140/<90 | | High BP | |  |  |  |
| **Variable** | n = 164 | | n = 236 | |  | p |  |
| Use of BP drug category, n (%) |  |  |  |  |  |  |  |
| ARB (C09CA) | 88 | (53.7) | 127 | (53.8) |  | 0.976 |  |
| Calcium channel blocker (C08C) | 74 | (45.1) | 91 | (38.6) |  | 0.190 |  |
| Beta blocker (C07AA,C07AB) | 57 | (34.8) | 78 | (33.1) |  | 0.723 |  |
| ACE inhibitor (C09AA) | 45 | (27.4) | 47 | (19.9) |  | 0.079 |  |
| Thiazide diuretic (C03AA) | 40 | (24.4) | 48 | (20.3) |  | 0.336 |  |
| Loop diuretic (C03C) | 9 | (5.5) | 8 | (3.4) |  | 0.306 |  |
| Alfa blocker (C02CA,G04CA01) | 6 | (3.7) | 11 | (4.7) |  | 0.625 |  |
| Aldosterone antagonist (C03DA) | 6 | (3.7) | 7 | (3) |  | 0.701 |  |
| Using lipid-lowering drugs, n (%) | 81 | (49.4) | 101 | (42.8) |  | 0.193 |  |
| ACE: Angiotensin converting enzyme; ARB: Angiotensin 2 receptor blocker; BP: blood pressure | | | | | | |  |
|  | | | | | | | |

|  | | | | | | | | | |
| --- | --- | --- | --- | --- | --- | --- | --- | --- | --- |
|  |  |  | Non-exercise activity | | | | | | |
|  |  |  | 1 | 2 | 3 | 4 | 5 | 6 | 7 |
|  |  | min* | 0 | <30 | 30–60 | 60–120 | 120–180 | 180–300 | >300 |
| Exercise | 1 | 0 | 3 | 4 | 5 | 6 | 7 | 8 | 9 |
|  | 2 | <30 | 5 | 6 | 7 | 8 | 9 | 10 | 11 |
|  | 3 | 30–60 | 7 | 8 | 9 | 10 | 11 | 12 | 13 |
|  | 4 | 60–120 | 9 | 10 | 11 | 12 | 13 | 14 | 15 |
|  | 5 | >120 | 11 | 12 | 13 | 14 | 15 | 16 | 17 |
| <11 = sedentary. ≥11 = achieved recommended physical activity.  *Minutes per week | | | | | | | | | |

| **Supplementary Figure 1.** Classification of activity level |
| --- |
|  |
